# Epidemiological features of hypertension among ischemic survivors in Northeast China: insights from a population-based study, 2017–2019

**DOI:** 10.1186/s12889-021-11692-x

**Published:** 2021-09-09

**Authors:** Li Jing, Yuanmeng Tian, Guocheng Ren, Limin Zhang, Lei Shi, Dong Dai, Liying Xing, Shuang Liu

**Affiliations:** 1 Department of Ultrasound, The Fourth Affiliated Hospital of China Medical University, Shenyang, Liaoning China; 2 Department of Chronic Diseases Control, Disease Control and Prevention of Liaoning Province, Shenyang, Liaoning China; 3 Institute of Preventive Medicine, China Medical University, Shenyang, Liaoning China; 4 Department of Cardiology, Central hospital of Chao Yang City, Chaoyang, Liaoning China; 5 Department of Cardiovascular Ultrasound, The First Hospital of China Medical University, Shenyang, Liaoning China; 6 Department of Chronic Diseases Control, Disease Control and Prevention of Liao Yang City, Liaoyang, Liaoning China; 7 Department of Chronic Diseases Control, Disease Control and Prevention of Dan Dong City, Dandong, Liaoning China

**Keywords:** Ischemic stroke, Hypertension, Epidemiological, China

## Abstract

**Background:**

Hypertension remains the major modifiable risk factor of stroke recurrence. The study aimed to determine the up-to-date epidemiological features of hypertension among the survivors of ischemic stroke.

**Methods:**

Our cross-sectional study included 18,796 adults aged ≥40 years and residing in northeast China. Ischemic stroke was diagnosed according to the World Health Organization’s criteria, which requires the clinical record, computed tomography (CT) and/or magnetic resonance imaging (MRI) during the hospital stay. Hypertension was defined according to the Chinese hypertension guidelines (mean SBP ≥140 mmHg and/or mean DBP ≥90 mmHg, and/or self-reported use of anti-hypertensive medication in the past 2 weeks).

**Results:**

Of the 986 survivors of ischemic stroke, 819 (83.1%) were identified with hypertension (535 were pre-stroke hypertension and 284 were post-stroke hypertension). Among hypertensive patients, the awareness and treatment rates were 76.8 and 66.7% respectively. Only 11.0% achieved an appropriate blood pressure (< 140 mmHg and < 90 mmHg) among those who took hypertensive medications. 16.8% of treated hypertensive patients received combination therapy, and calcium channel blockers were the most frequently used anti-hypertensive medication as monotherapy. The mean systolic blood pressure (SBP) and diastolic blood pressure (DBP) of the stroke population was 155.3 ± 22.9 mmHg and 89.2 ± 12.3 mmHg. Both SBP and DBP were higher in rural patients than in urban patients (158.5 ± 23.8 mmHg vs. 146.4 ± 17.5 mmHg and 90.3 ± 12.9 mmHg vs. 85.9 ± 10.1 mmHg, respectively; *p* < 0.001). The rates of stage 2 and above hypertension in the ischemic stroke population were 32.5 and 18.7%, and was significantly higher in rural areas than in urban areas.

**Conclusions:**

The prevalence of poorly-controlled hypertension and the high rates of blood pressures at stages 2 and above in patients with prior ischemic stroke demonstrated an alarming situation in northeast China.

## Background

Stroke has emerged as one of the major causes of death and disability in the world, with more than 80% of strokes worldwide occurring in low- and middle-income countries [1]. In China, where one-fifth of the world’s population resides, more than 2 million people are affected annually by stroke, contributing to a high disability-adjusted life-years (DALY) loss [2, 3]. However, the burden of stroke in China continues to increase due to an aging population, cultural changes in life-style, and a high prevalence and poor management of related risk factors [3]. In China, stroke was responsible for nearly 1.5 million premature deaths in 2017, the years of life lost (YLLs) caused by stroke has increased 14.6% from 1990 to 2017 [4]. Therefore, stroke continues to be a major public healthcare challenge in China.

More than 70% of the stroke survivors had an ischemic stroke according to our previous study [5], which was associated with a high morbidity rate due to the emotional and social consequences of the experience, in addition to a high risk for stroke recurrence and cardiovascular comorbidities. A previous study showed that the cumulative risk of recurrence within the first 30 days after an initial stroke was 3% but soared to 40% in the first 10 years [6]. Patients with recurrent strokes were more likely to have worse functional disabilities than those with only one stroke [7]. Therefore, stroke recurrence remains an ongoing health concern and secondary prevention must be emphasized.

Hypertension has long been considered the most common and important potentially modifiable risk factor for recurrent vascular events and is strongly associated with a worse prognosis [8]. Previous studies have consistently confirmed that the management of hypertension could significantly reduce the likelihood of stroke recurrence and long-term adverse outcomes in patients with a history of ischemic stroke [9]. In our previous study, we found that hypertension was prevalent in rural northeast China’s stroke population [7]; however, detailed information regarding this remains unclear. In the present study, we conducted a cross-sectional survey to profile the current hypertensive status of the ischemic stroke population for future research on secondary prevention strategies in northeast China.

## Methods

### Study population and design

**Fig. 1:**
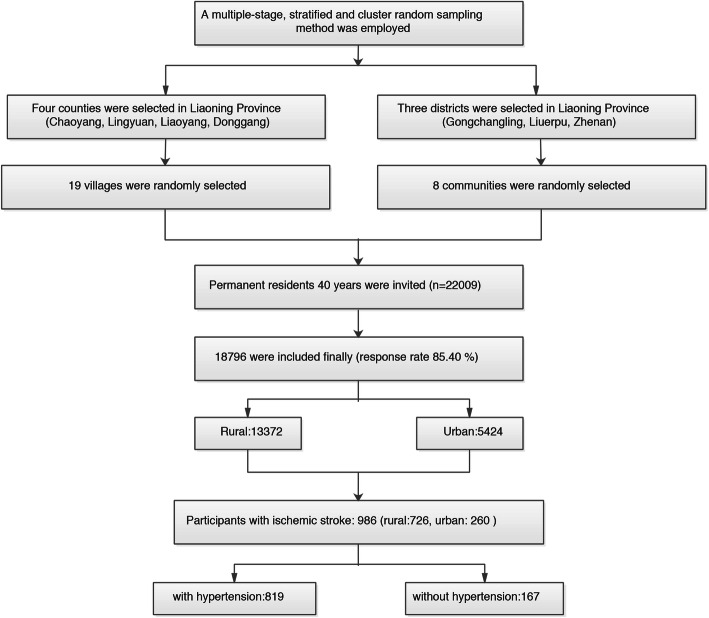
Flowchart of population selection

This cross-sectional study was undertaken between September 2017 and March 2019 in northeast China. To ensure the samples were representative, a multi-stage, stratified, and cluster random sampling method was employed. Four rural counties (Chaoyang, Lingyuan, Liaoyang, and Donggang) and three urban districts (Gongchangling, Liuerpu, and Zhenan) were randomly selected from Liaoning Province. In total, 19 rural villages and eight communities were randomly selected from these four counties and three cities. All permanent residents (individuals registered in the Local Household Register System and living in the selected communities for ≥6 months when the study began) [10] aged ≥40 years in each village and community (*n* = 22,009) were eligible to participate, except those who were pregnant or had a mental disorder; in total, 18,796 (85.4%) participants ultimately completed the study (Fig. 1). The study was granted approval by the Central Ethics Committee at the China National Center for Cardiovascular Disease (Beijing, China). Written informed consent was obtained from all participants [7, 11].

The prevalence time was determined as August 31, 2017, while new onset stroke cases during the survey were excluded in the calculation of stroke prevalence. Participants with a doubtful or certain history of stroke were further confirmed by well-trained neurologists in accordance with the World Health Organization recommendations. The clinical record, computed tomography (CT) and/or magnetic resonance imaging (MRI) during the hospital stay were required to make the diagnosis of ischemic stroke [12]. The data collection and measurement strategy used has been described previously [7, 11, 13].

After at least 5 min of rest in a seated position in the morning, systolic blood pressure (SBP) and diastolic blood pressure (DBP) were measured three times at 2-min intervals using a standardized automatic electronic sphygmomanometer (J30; Omron, Kyoto, Japan). Hypertension was diagnosed according to the following Chinese hypertension guidelines: a mean SBP ≥140 mmHg and/or a mean DBP ≥90 mmHg and/or self-reported use of antihypertensive medication in the past 2 weeks [14]. Stage 1 hypertension was defined as SBP 140–159 mmHg and/or DBP 90–99 mmHg; stage 2 as SBP 160–179 mmHg and/or a DBP 100–109 mmHg; and stage 3 as SBP ≥180 mmHg and/or DBP ≥110 mmHg [11]. In patients with stroke, awareness of hypertension was defined as the participants clearly indicated that they were diagnosed with hypertension by a certified doctor. The treatment for hypertension was defined as the use of an antihypertensive medication in the past 2 weeks. Control of hypertension was defined as an average SBP < 140 mmHg and an average DBP < 90 mmHg [15].

Medications were classified as thiazide-type diuretics, calcium channel blockers, *β*- blockers, angiotensin-converting enzyme inhibitors, angiotensin II receptor blockers and others such as Chinese medical and vasodilators. Patients who used ≥2 drugs were defined as receiving combination therapy; those using only one drug as monotherapy.

### Statistical methods

Descriptive statistics were calculated for all variables. Continuous variables with normal distribution are reported as means and standard deviations, and compared using one-way analysis of variance. Differences between groups were compared using a χ^2^ test for categorical variables. Pre-stroke and post-stroke hypertension were calculated according to the diagnostic time of stroke and hypertension. All statistical analyses were conducted using SPSS22.0 (SPSS Inc., Chicago, IL, USA); *P* values < 0.05 were considered statistically significant.

## Results

### Characteristics of the population

The characteristics of the stroke population are shown in Table 1. Overall, 986 participants had a diagnosis of ischemic stroke (Fig. 1). These participants included 495 men (50.2%) and 491 women (49.8%) whose average age was 66.3 ± 8.4 years. A total of 62.0% had a primary school education or less, 46.9% were low-socioeconomic participants with an annual household income < 5000 yuan (approximately $700).

**Table 1:** Characteristics of the 986 ischemic stroke patients

Characteristics	Region		Sex		Total	P for region	P for sex
	Urban	Rural	Men	Women			
**Stroke, n (%)**	260 (26.4)	726 (73.6)	495 (50.2)	491 (49.8)	986		
Mean age, year	67.1 ± 8.0	65.9 ± 8.5	66.2 ± 8.8	66.3 ± 8.0	66.3 ± 8.4	0.647	0.539
40–49	4 (1.5)	23 (3.2)	17 (3.4)	10 (2.0)	27 (2.7)		
50–59	45 (17.3)	124 (17.1)	84 (17.0)	85 (17.3)	169 (17.1)		
60–69	113 (43.5)	326 (44.9)	225 (45.5)	214 (43.6)	439 (44.5)		
70–79	84 (32.3)	220 (30.3)	144 (29.1)	160 (32.6)	304 (30.8)		
> =80	14 (5.4)	33 (4.5)	25 (5.1)	22 (4.5)	47 (4.8)		
**Education, n (%)**						< 0.001	< 0.001
Primary school or lower	110 (42.3)	501 (69.0)	254 (51.3)	357 (72.7)	611 (62.0)		
Middle school	110 (42.3)	181 (24.9)	181 (36.6)	110 (22.4)	291 (29.5)		
High school or above	40 (15.4)	44 (6.1)	60 (12.1)	24 (4.9)	84 (8.5)		
**Annual household income, n (%)**						< 0.001	0.033
< 5000 (yuan)	17 (6.5)	445 (61.3)	224 (45.3)	238 (48.5)	462 (46.9)		
5000–9999 (yuan)	38 (14.6)	149 (20.5)	88 (17.8)	99 (20.2)	187 (19.0)		
10,000–19,999 (yuan)	42 (16.2)	70 (9.6)	51 (10.3)	61 (12.4)	112 (11.4)		
> =20,000 (yuan)	163 (62.7)	62 (8.5)	132 (26.7)	93 (18.9)	225 (22.8)		

### Blood pressure levels among ischemic stroke survivors

**Fig. 2:**
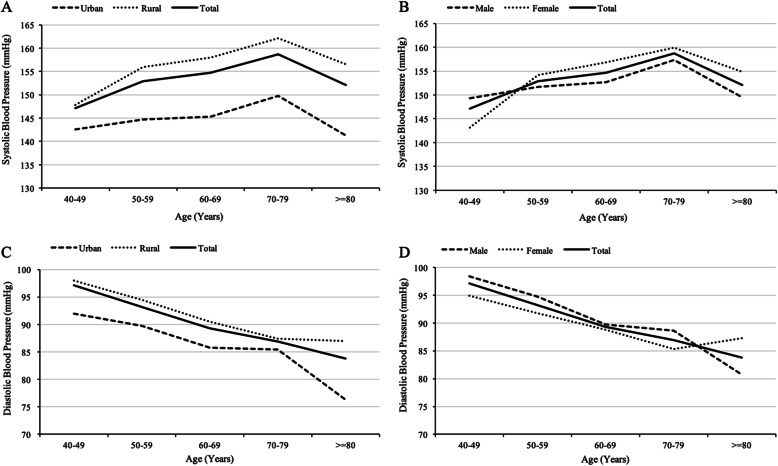
Blood pressure levels among ischemic stroke survivors in northeast China by region (A: systolic blood pressure, C: diastolic blood pressure) and sex (B: systolic blood pressure, D: diastolic blood pressure)

The mean SBP and DBP in the ischemic stroke population were 155.3 ± 22.9 mmHg and 89.2 ± 12.3 mmHg, respectively and were significantly higher in rural residents than in urban residents (158.5 ± 23.8 mmHg vs. 146.4 ± 17.5 mmHg and 90.3 ± 12.9 mmHg vs. 85.9 ± 10.1 mmHg, respectively; *p* < 0.001). The mean SBPs were higher in women patients than in men patients (157.0 ± 23.4 mmHg vs. 153.6 ± 22.3 mmHg, respectively; *p* = 0.019); however, the DBP levels in the men ischemic stroke population was higher than that of women (90.1 ± 12.0 mmHg vs. 88.2 ± 12.6 mmHg, respectively; *p* = 0.018) (Fig. 2).

### Prevalence of hypertension among the ischemic stroke population

Among the ischemic stroke population, 819 participants had been identified with hypertension (535 were pre-stroke hypertension and 284 were post-stroke hypertension), as shown in Table 2. The overall prevalence of hypertension was 83.1% and was higher in women than in men (85.7% vs. 80.4% respectively; *p* = 0.025); however, no significant difference was found between urban and rural residents (80.0 vs. 84.2% respectively; *p* = 0.125) (Table 2).

**Table 2:** Prevalence of hypertension among 986 ischemic stroke survivors in northeast China

Characteristics	N	Region		Sex		Total	P for region	P for sex
		Urban	Rural	Men	Women			
40–49	22	100.0	78.3	70.6	100.0	81.5	0.302	0.057
50–59	133	73.3	80.6	78.6	78.8	78.7	0.305	0.968
60–69	358	79.6	82.2	77.8	85.5	81.5	0.545	0.037
70–79	269	85.7	89.5	88.2	88.8	88.5	0.349	0.88
> = 80	37	64.3	84.8	72.0	86.4	78.7	0.115	0.23
Overall	819	80.0	84.2	80.4	85.7	83.1	0.125	0.025

**Fig. 3:**
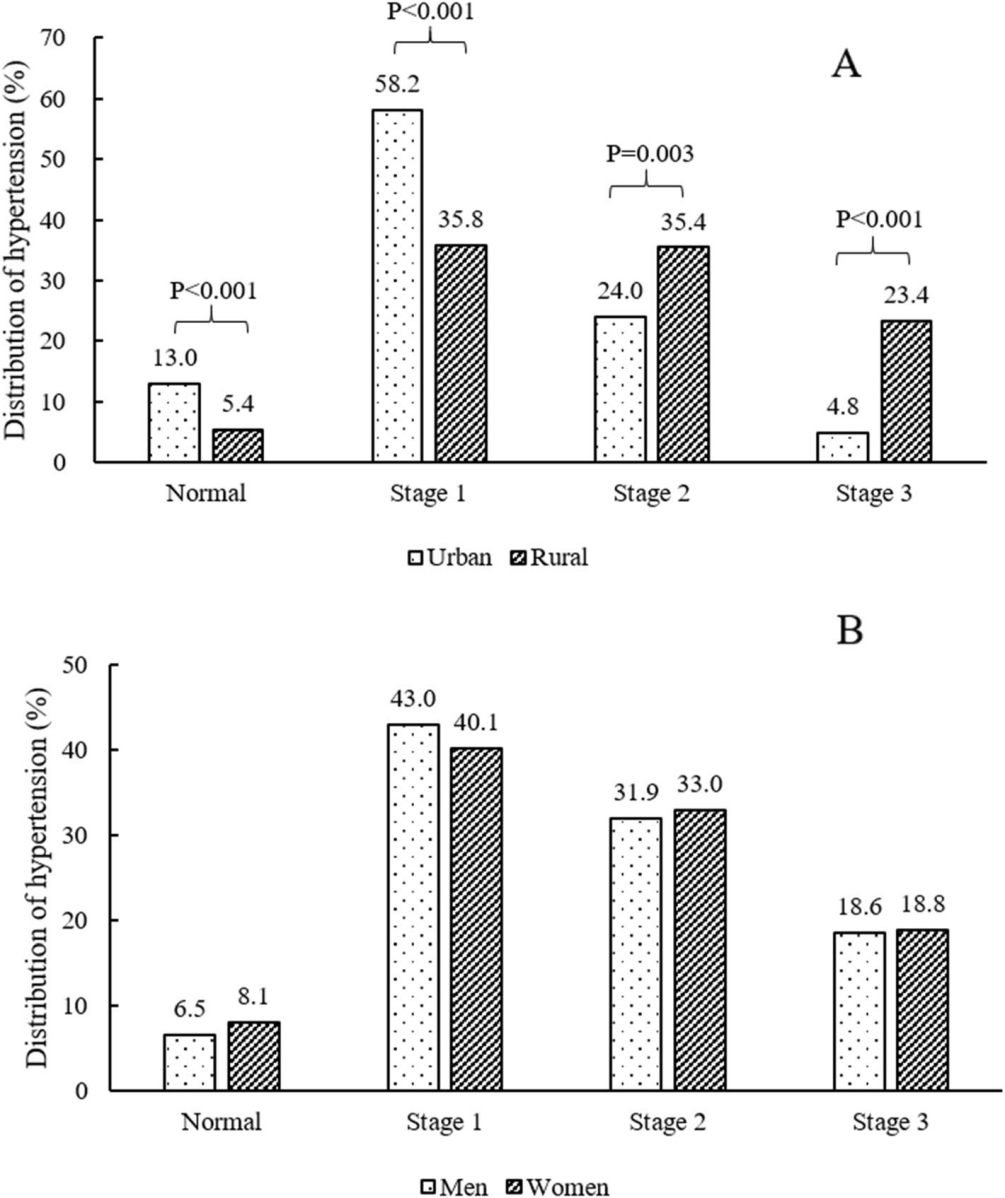
The distribution of hypertension in ischemic stroke survivors by region (A) and sex (B)

The prevalence of stages 1, 2, and 3 hypertension among hypertensive patients with ischemic stroke were 41.5, 32.5, and 18.7%, respectively. The prevalence of stages 2 and 3 hypertension were significantly higher in rural areas than in urban areas (24.0% vs. 35.4 and 4.8% vs. 23.4%, respectively) (Fig. 3).

### Awareness, treatment, and control of hypertension among the ischemic stroke population

Among the ischemic stroke patients with hypertension, the awareness and treatment rates were 76.8 and 66.7%, respectively. Among those who were taking anti-hypertensive drugs, only 11.0% of treated hypertensive patients had a blood pressure lower than 140/90 mmHg (Table 3).

**Table 3:** Awareness, treatment and control of hypertension among 986 ischemic stroke survivors in northeast China (%)

Characteristics	Awareness	Treatment^**a**^	Control^**b**^
**Overall**	629 (76.8)	546 (66.7)	60 (11.0)
**Age Group**			
40–49	16 (72.7)	13 (59.1)	0 (0.0)
50–59	108 (81.2)	98 (73.7)	7 (7.1)
60–69	278 (77.7)	242 (67.6)	31 (12.8)
70–79	201 (74.7)	170 (63.2)	16 (9.4)
> =80	26 (70.3)	23 (62.2)	6 (26.1)
*P* value	0.503	0.244	0.041
**Sex**			
Men	294 (73.9)	243 (61.1)	26 (10.7)
Women	335 (79.6)	303 (72.0)	34 (11.2)
*P* value	0.053	0.001	0.846
**Education,n(%)**			
Primary school or lower	403 (76.9)	349 (66.6)	40 (11.5)
Middle school	176 (76.2)	153 (66.2)	14 (9.2)
High school or above	50 (78.1)	44 (68.8)	6 (13.6)
*P* value	0.944	0.93	0.628
**Annual household income,n(%)**			
< 5000 (yuan)	300 (74.8)	256 (63.8)	20 (7.8)
5000–9999 (yuan)	115 (78.2)	99 (67.3)	14 (14.1)
10,000–19,999	73 (79.3)	61 (66.3)	9 (14.8)
> =20,000 (yuan)	141 (78.8)	130 (72.6)	17 (13.1)
*P* value	0.619	0.227	0.164
**Region**			
Urban	159 (76.4)	149 (71.6)	27 (18.1)
Rural	470 (76.9)	397 (65.0)	33 (8.3)
*P* value	0.887	0.078	0.001

Treatment^a^ indicated the treatment rate among the overall hypertensive patients with prior ischemic stroke; Control^b^ indicated the control rate among those who took anti-hypertensive medications

The rates of awareness and treatment were higher in women than in men (79.6% vs. 73.9%, *p* = 0.053; and 72.0% vs. 61.1%, *p* = 0.001; respectively); however, blood pressure control was not significantly different between women and men (11.2% vs. 10.7%, respectively; *p* = 0.846). Although the awareness rate was similar in urban and rural residents, stroke survivors from urban areas had better control of their hypertension compared to those from rural areas (18.1% vs. 8.3%, respectively; *p* = 0.001).

**Fig. 4:**
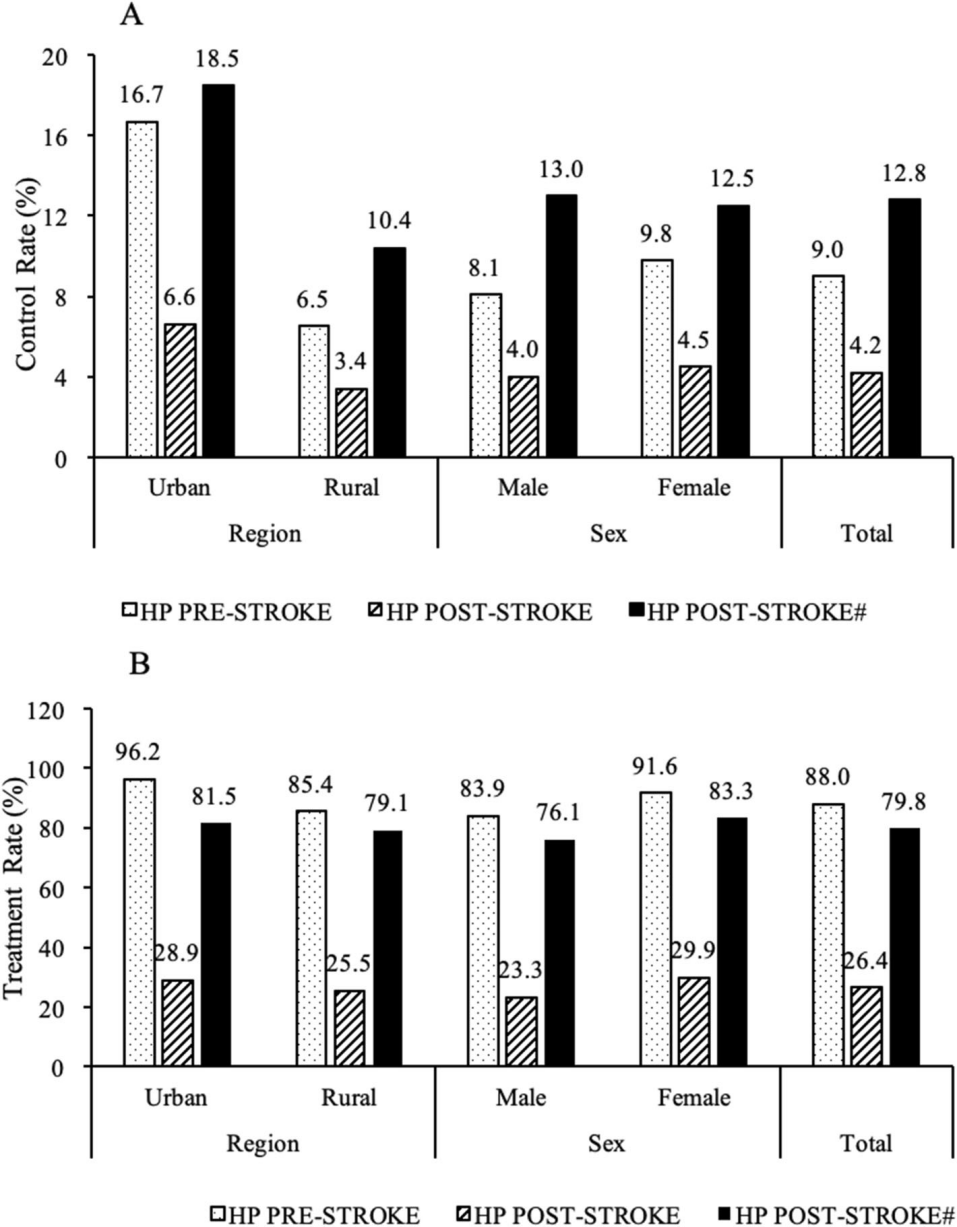
Treatment (B) and control (A) of hypertension according to the stroke index time by region and sex. HP pre-stroke: presence of hypertension among pre- stroke patients; HP post-stroke: presence of hypertension among post- stroke patients; HP post-stroke^#^: presence of hypertension among stroke patients after excluded newly diagnosed hypertension during the survey period

Among the ischemic stroke patients with hypertension, after excluded 190 newly identified hypertensive patients during the survey period, 535(65.3%) had a history of hypertension before the index stroke, 88.0% of whom were taking anti-hypertensive medicine, but the control rate was only 9.0%. Ninety-four patients (11.5%) had hypertension identified after the index stroke, the treatment and control rates were 79.8 and 12.8%, respectively (Fig. 4).

### Anti-hypertensive medicine use

Among 546 treated patients, 83.2% of prescriptions were for one medication, 16.8% were two or more medications. The control rates among patients taking monotherapy and combination therapy were 11.2 and 9.8%, respectively. The most frequently used anti-hypertensive medication as monotherapy were calcium channel blockers (47.4%), however, the control rate among those patients was only 9.8% (Table 4).

**Table 4:** Antihypertensive medicine use and the control rate of hypertension among 546 anti-hypertensive treatments patients

	n	Percentages (%)	Control (n, %)
Monotherapy*	454	83.2	51 (11.2)
TDs	19	4.2	6 (31.6)
CCBs	215	47.4	21 (9.8)
BBs	5	1.1	1 (20.0)
ACEIs	28	6.2	2 (7.1)
ARBs	42	9.3	4 (9.5)
Other	145	31.9	17 (11.7)
**Combination therapy**	92	16.8	9 (9.8)

*Abbreviations*: *TDs* thiazide-type diuretics, *CCBs* calcium channel blockers, *BBs* β-blockers, *ACEIs* angiotensin-converting-enzyme inhibitors, *ARBs* angiotensin receptor blockers

## Discussion

Our study revealed an astonishing prevalence of hypertension among the ischemic stroke population in northeast China. The high prevalence and low management of hypertension in this population combine to make ischemic stroke a considerable burden. Additionally, the high rates of stages 2 and above hypertension show that there is a substantial underlying risk of adverse cardiovascular outcomes in patients with ischemic stroke, particularly in those who have settled in rural areas. Therefore, the secondary prevention of stroke, especially long-term follow-up and management of risk factors including hypertension, is critically important for improving the prognosis in this population and should be emphasized.

Expert consensus guidelines recommend that the recognition and control of blood pressure is essential in stroke survivors to avoid adverse cardiovascular events such as stroke recurrence [16]. In the present study, we found the prevalence of hypertension among patients with prior ischemic stroke to be 83.1%, which was higher than that reported by the United States national survey (72.2–74.4%) [17, 18], while the rate of hypertension in the general population of northeast China was 56.8% [11], indicating high stroke burdens in those population.

The mean SBP and DBP levels in our patient cohort were 155.3 ± 22.9 mmHg and 89.2 ± 12.3 mmHg, respectively, which were significantly higher than that of the general population in Northeast China (142.9 ± 22.6 mmHg and 85.4 ± 11.6 mmHg) [11]. Noticeably, rural patients had a significantly higher blood pressure at stage 2 and above compared to urban patients. One study revealed that a reduction in diastolic blood pressure of 5 mmHg could reduce the risk of stroke by one third [19]. High systolic blood pressure (> 140 mmHg) has also been shown to be correlated to an increased risk of recurrent stroke [20]. Stroke recurrence could significantly worsen functional disabilities and increase health care costs, therefore, management of hypertension in stroke survivors could have substantial benefits, especially in rural areas.

The question of whether anti-hypertensive treatments significantly decrease the rate of recurrent stroke remains somewhat controversial. Anti-hypertensive medication use in patients with high baseline blood pressures have been shown to reduce all-cause and, specifically, cardiovascular mortalities; however, aggressive blood pressure reduction has also been shown to worsen outcomes in stroke patients with preexisting cardiovascular diseases [17, 21]. A previous study determined that a very low-normal SBP level (< 120 mmHg) and high SBP (≥140 mmHg) were both associated with an increased risk of stroke recurrence [20]. Therefore, in the present study, hypertension control was defined as SBP < 140 mmHg and DBP < 90 mmHg. In addition, we identified that up to 65.3% had a history of hypertension before the index stroke, but only 9.0% of them were controlled. Even in patients with hypertension identified after the index stroke, the control rate remained extremely low (12.8%), especially in women and rural residents, and a large number of stroke patients still had uncontrolled hypertension, indicated that secondary stroke prevention was not sufficiently effective in northeast China.

Several large trials have recommended anti-hypertensive treatment for patients with a history of stroke to prevent future vascular events [19]. Previous studies indicated that most hypertensive patients with comorbidities need more than on single anti-hypertensive medication [22]. However, in our present study cohort, 83.2% of the hypertensive patients with medical treatment were using monotherapy currently, even in those with combination therapy, the blood pressures were poorly controlled. Overall, the rates of treatment and adequate blood pressure control among ischemic stroke survivors in the present study remained unacceptably low, especially since only 11.0% of the patients taking anti-hypertensives were at their target blood pressures.

Moreover, adequate blood pressure control in rural areas was worse than that in urban areas, and the prevalence of stages 2 and 3 hypertension were significantly higher in rural survivors than in urban survivors, probably due to the relatively low socioeconomic status and limited access to necessary health care in rural areas [23]. Previous study indicated that rural population tended to have relatively low educational levels [23]. In addition, with the rapid economic progression, particularly in rural area, urbanization is associated with potential life style changes, which might further lead to increase in coexisting risk factors, including diabetes, dyslipidemia, obesity and alcohol consumption [24]. Therefore, secondary prevention in rural stroke populations is relatively more difficult and crucial.

Furthermore, we found women tended to have better control of hypertension compared to men, possibly because women were more sensitive to health education and had better compliance rates [25]. Since a large percentage of the stroke population we studied had uncontrolled hypertension, especially in men, indicated those population is lagging in risk management, recurrent stroke is an important concern in the coming decades.

Ischemic stroke remains an important healthcare challenge because of its increasing prevalence, high percentage of recurrence, and disabling sequelae [26]. In recognition of the high mortality, increased disability, and greater health care costs of recurrent stroke, reducing the prevalence and recurrence of the disease should be emphasized. Our previous study found that individuals experiencing recurrent stroke were more likely to have worse functional disabilities [7].

The present study comprehensively profiled the status of hypertension among ischemic stroke survivors in northeast China, providing population-based evidence for formulating comprehensive strategies for secondary stroke prevention and care in those areas. Our study has several limitations. Firstly, since it is a cross-sectional survey, we only have data on blood pressure at one point in time and further study focusing on the relationship between hypertension and long-term outcomes among the stroke population should be undertaken. Secondly, since the prevalence of hypertension was our only focus, other risk factors and comorbidities were not assessed in this study. Thirdly, our study did not include medical compliance in the present study, further implementation studies will need to account for anti-hypertensive medical adherence. Lastly, we could not analyze the duration of hypertension and time passed since stroke because of memory loss in stroke patients.

## Conclusions

Our study revealed some alarming information about hypertension among patients with prior ischemic stroke in northeast China. The high prevalence and poor control in addition to the high rate of stages 2 and above hypertension all indicate a large stroke burden in this population, especially in rural areas. Therefore, interventions tailored to improve the control of hypertension among this high-risk population should be undertaken.

## Data Availability

The datasets generated for and analyzed in the study are not publicly available due to China Medical University’s privacy policy, but are available from the corresponding author upon reasonable request.

## Abbreviations

SBP: Systolic blood pressure
DBP: Diastolic blood pressure
